# Virtual randomized study comparing lobectomy and particle beam therapy for clinical stage IA non-small cell lung cancer in operable patients

**DOI:** 10.1093/jrr/rrab060

**Published:** 2021-07-05

**Authors:** Young-Seok Seo, Woo-Yoon Park, Si-Wook Kim, Dohun Kim, Byung Jun Min, Won-Dong Kim

**Affiliations:** Department of Radiation Oncology, Chungbuk National University Hospital, Cheongju 28644, Korea; Department of Radiation Oncology, Chungbuk National University Hospital, College of Medicine, Chungbuk National University, Cheongju 28644, Korea; Department of Thoracic and Cardiovascular Surgery, Chungbuk National University Hospital, College of Medicine, Chungbuk National University, Cheongju 28644, Korea; Department of Thoracic and Cardiovascular Surgery, Chungbuk National University Hospital, College of Medicine, Chungbuk National University, Cheongju 28644, Korea; Department of Radiation Oncology, Chungbuk National University Hospital, Cheongju 28644, Korea; Department of Radiation Oncology, Chungbuk National University Hospital, College of Medicine, Chungbuk National University, Cheongju 28644, Korea

**Keywords:** lung cancer, lobectomy, particle beam therapy (PBT)

## Abstract

To the best of our knowledge there have been no randomized controlled trials comparing lobectomy—a standard treatment for patients with early-stage non-small cell lung cancer (NSCLC)—and particle beam therapy (PBT), the best performing existing radiotherapy. We conducted a virtual randomized trial in medically operable patients with stage IA NSCLC to compare lobectomy and PBT effectiveness. A Markov model was developed to predict life expectancy after lobectomy and PBT in a cohort of patients with stage IA NSCLC. Ten thousand virtual patients were randomly assigned to each group. Sensitivity analyses were performed as model variables and scenarios changed to determine which treatment strategy was best for improving life expectancy. All estimated model parameters were determined using variables extracted from a systematic literature review of previously published articles. The preferred strategy differed depending on patient age. In young patients, lobectomy showed better life expectancy than that of PBT. The difference in life expectancy between lobectomy and PBT was statistically insignificant in older patients. Our model predicted lobectomy as the preferred strategy when operative mortality was under 5%. However, the preferred strategy changed to PBT if operative mortality post lobectomy was over 5%. For medically operable patients with stage IA NSCLC, our Markov model revealed the preferred strategy of lobectomy or PBT regarding operative mortality changed with varying age and comorbidity. Until randomized controlled trial results become available, we hope the current results will provide a rationale background for clinicians to decide treatment modalities for patients with stage IA NSCLC.

## INTRODUCTION

Lung cancer is the leading cause of cancer-related deaths worldwide [1]. With the development and widespread use of low-dose computed tomography for lung cancer screening, the likelihood of finding small nodules in the lungs has increased. Recently, application of these clinical adaptations to individuals at high-risk of developing lung cancer has led to an increase in the number of early-stage non-small cell lung cancer (NSCLC) diagnoses [2–4]. In operable patients with early-stage NSCLC, lobectomy and mediastinal lymph node dissection have been performed as a standard treatment [5]. Nevertheless, stereotactic body radiation therapy (SBRT) with photons has been suggested as an alternative treatment choice for patients who are medically inoperable due to severe comorbidity or poor pulmonary function [6]. Based on excellent clinical outcomes, SBRT has been widely applied in clinical practice to inoperable patients with early-stage NSCLC. Although SBRT has not yet been directly compared to that of standard surgery in a randomized trial, several studies have shown the efficacy of SBRT is potentially comparable to lobectomy in select patient groups, such as those with stage I NSCLC [7–9].

As technology advances, radiotherapy techniques using particle beam therapy (PBT)—such as protons and carbon ions—have emerged showing the physical and biological benefits are comparable to photons used in traditional radiotherapy. The Bragg peak of PBT allows deep-seated tumors in the body to be irradiated with a very sharp dose gradient at the distal edge of the tumor target, resulting in a physically superior dose distribution compared to that of photons [10, 11]. In addition, because of higher linear energy transfer, greater relative biological effectiveness and a lower oxygen enhancement ratio, carbon ions can provide potential biologic advantages compared to that provided by photons. As a result, PBT is able to increase the probability of tumor control [12, 13]. Recently, a meta-analysis including 72 SBRT studies and nine PBT studies for early-stage NSCLC reported the superior therapeutic effect of PBT [14].

To the best of our knowledge there have been no randomized controlled trials comparing the results of lobectomy—a standard treatment for patients with early-stage NSCLC—and PBT, which is known to exhibit the best performance among existing radiotherapy. PBT is not yet widely available and its costs are high. As a result, there are only a few centers around the world where PBT is available. Not only does this limit the availability of the treatment to patients, it also makes it difficult to conduct randomized controlled trials comparing PBT and surgical interventions. To overcome these practical limitations, we conducted a virtual randomized trial in virtual medically operable patients with stage IA NSCLC and compared the effectiveness of lobectomy and PBT using Markov model analysis. A Markov model is a computerized model widely used in the study of cost-effectiveness analysis and it can be used to simulate the effects of competing interventions and identify key variables that may affect the outcomes of therapeutic strategies.

## MATERIALS AND METHODS

### Selection criteria and scenario composition

**Fig. 1.**
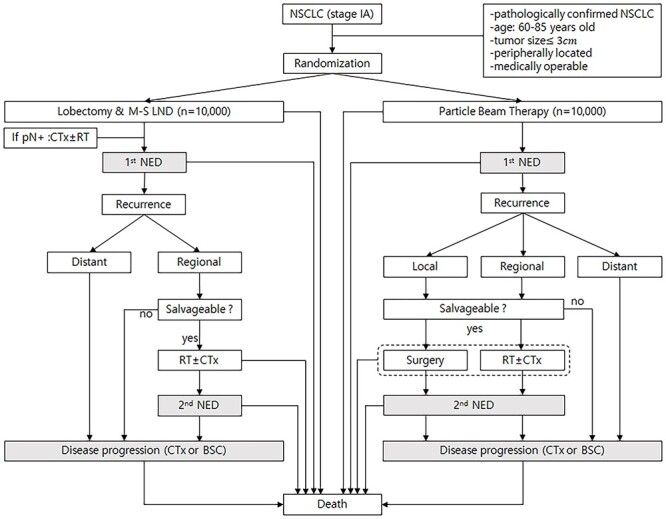
Scenario for the Markov state transition model of stage IA NSCLC**.** Each rectangle represents a state of health. From the initial state, patients are randomized to undergo lobectomy or PBT. Straight arrows represent the changes that may occur during each cycle or a very short time interval. In contrast, gray rectangles mean that the patients may remain in the same Markov state for more than one cycle. CTx; chemotherapy, RT; conventional radiotherapy, pN+; pathologically positive lymph node, NED; no evidence of disease, BSC; best supportive care.

Inclusion criteria for the virtual study was as follows: (i) pathologically-confirmed NSCLC, (ii) stage IA according to the *American Joint Committee on Cancer Staging Manual* 8th Edition (*AJCC* 8^th^), (iii) peripherally located tumor, (iv) medically operable patients. A Markov model was developed to predict the remaining life expectancy after lobectomy or PBT in a cohort of virtual patients with stage IA NSCLC (Fig. 1). The age of patients enrolled in this model was limited to 60- to 85 years of age at the time of diagnosis and the patients were stratified into six groups based on five-year windows of age at diagnosis (60, 65, 70, 75, 80 and 85 years of age).

Ten thousand virtual patients were randomly assigned to each of the lobectomy and PBT groups. In the Markov model, a virtual patient could potentially go through a total of 23 states of health from the time of receiving each treatment to death (Fig. 1). The probability of transition from each state of health to the next was defined by parameters extracted from a literature review (see Table 1). Until death, the patients could stay in three Markov states (gray rectangles in Fig. 1). The first state was no evidence of disease after primary treatment, the second state was no evidence of disease after salvage treatment for recurrence, and the third state was a state of disease progression. The patients could move to other Markov states every year, and this is defined as one cycle. The patients could stay longer than one cycle in three Markov states. Half-cycle correction was used under the assumption that each transition occurred halfway during the cycle. To set the follow-up period after treatment to 15 years, it was assumed that the Markov model was repeated up to 15 cycles. Commercially available TreeAge Pro software (TreeAge Software, Williamstown, MA) was used to generate the Markov model.

**Table 1:** Estimated values of the variables used for the Markov model extracted from the literature

**Variables**		**Lobectomy**	**Particle beam therapy**
Annual mortality of general population in those aged 70		0.018679 [59]	
Procedure-related mortality in those aged 70		0.0295	0.001(0–0) [19, 20, 43–45, 60–62]
Procedure-related mortality with conventional radiotherapy		0.0010 [63]	
One year probability of disease progression		0.0400 (0.0133–0.0742) [31–34, 64–69]	0.0568(0.0374–0.0723) [19, 20, 43–45, 61]
Rate of local failure only/total recurrence		0	0.0843(0–0.2222) [19, 20, 43, 45, 61]
Rate of loco-regional failure/total recurrence		0.2784 (0.0952–0.4) [5, 33, 65, 68–71]	0.1076(0–0.25) [19, 20, 43, 45, 61]
Probability of radical salvage treatment after recurrence	In local failure		0.6321 [19, 20]
	In regional failure	0.3446 (0.3172–0.3888) [21, 22]	0.3095(0.2941–0.6321) [17–19]
	In distant failure	0	0
One year probability of disease progression after radical salvage treatment	In local failure		0.0459(0–0.0799) [20, 23–26]
	In regional failure	0.2639 (0.2342–0.2865) [27–29]	0.3115 (0.254–0.3835) [18, 30]
Annual mortality of progressive disease (chemotherapy or best supportive care)		0.6268(0.4624–0.8105) [72–84]	

### Literature search strategy and data extraction

All estimated parameters used in the Markov model were determined using variables extracted from a systematic literature review of previously published articles, which are summarized in Table 1. The systemic review of the literature was performed using PubMed and all articles published as abstracts or full papers in English from January 2000 to October 2020 in peer-reviewed journals that assessed a survival benefit or tumor response after lobectomy or PBT as a primary treatment for stage IA NSCLC were selected. To identify all potentially relevant studies, we combined the search terms (‘NSCLC’ or ‘non-small cell lung cancer’) and (‘particle beam therapy’ or ‘proton therapy’ or ‘carbon ion therapy’ or ‘heavy ion therapy’) and (‘surgery’ or ‘lobectomy’). The types of surgery were not restricted by the technique, such as open thoracotomy, video-assisted thoracoscopic surgery (VATs) or robot-assisted thoracoscopic surgery (RATs). The literature pertaining to PBT with centrally located tumor or low biologically equivalent dose (BED) of less than 90 Gy was excluded. To calculate an overall representative value for each component, each outcome in the average was weighed by the number of patients in the articles.

### Summary of parameters and assumptions

Dissection of mediastinal lymph nodes was performed in the lobectomy group and patients with pathologically confirmed regional lymph node involvement were treated with adjuvant chemotherapy with or without radiotherapy. Procedure-related mortality of performing the lobectomy was extracted as the 90-days mortality rate according to the findings presented in the study of Stoke *et al.* [15]. For the virtual age group over 85-years-old, which was not suggested in the study by Stoke, we assumed a mortality rate by extrapolation. As no papers among those regarding PBT results for peripherally located NSCLC reported procedure-related death, we assumed the treatment-related mortality rate of PBT was equal to 0.001, regardless of age.

It was assumed there was no local recurrence in the lobectomy group and regional recurrence was defined as recurrence at the bronchial stump, ipsilateral hilar or mediastinal lymph node. In the PBT group, regional recurrence was defined as recurrence at the ipsilateral hilar or mediastinal lymph node. During follow-up, some patients with only loco-regional recurrence and without distant metastasis were considered candidates for salvage treatment. Various treatment modalities can be considered as salvage treatment for loco-regional recurrence; however, for simplification of the Markov model, it was assumed that lobectomy would be performed for local recurrence after PBT and conventional radiotherapy with or without chemotherapy would be performed for regional recurrence after PBT or lobectomy. In the both groups it was assumed that patients among the those with recurrences who did not receive salvage treatment moved directly to a state of disease progression (Fig. 1).

In an actual clinical setting, salvage treatment, such as surgery or radiotherapy, may be performed for oligometastases or secondary lung cancer. Our tumor board determined the likelihood of it affecting the outcome would be low as the probability of occurrence would be similar in both groups. The reported probability is a very wide range and a precise value is unclear [9, 16]. As a result, it was assumed that all patients with distant metastasis moved directly to the state of disease progression, without considering salvage treatment. Furthermore, the occurrence of secondary lung cancer in this scenario was not considered in order to simplify the Markov model.

Based on the literature, salvage treatment is performed in approximately 29–63% of patients with loco-regional recurrence who have been treated with lobectomy or PBT for primary NSCLC [17–22]. Although more than two repetitive salvage treatments are theoretically possible in cases of loco-regional recurrence, no studies were found that reported repeated salvage treatment after loco-regional recurrence in NSCLC [18, 23–30]. Considering the clinical reality, the number of repetitions of salvage treatment in patients with local recurrence was limited to one in the Markov model.

### Sensitivity analysis

As the variables and scenarios used in the model changed, sensitivity analysis was performed to determine which treatment was the best strategy for improving life expectancy. Assuming the values of the other parameters were constant, a one-way sensitivity analysis was performed to analyze the effect on life expectancy with only a single parameter of interest being changed. A two-way sensitivity analysis was performed to evaluate the effect on life expectancy when two parameters of interest were changed. To consider the uncertainty associated with parameter estimation, we performed a second-order Monte Carlo probabilistic sensitivity analysis.

### Validation of the Markov model

To confirm the reliability of the Markov model, we compared the survival rates predicted by our Markov model with actual existing clinical results of survival rates reported after lobectomy or PBT in patients with operable stage IA NSCLC. Curves of five-year overall survival rates predicted using our model were plotted and the five-year overall survival rate reported in the actual clinical results were marked on the survival curves.

### Tumor board organization

A tumor board composed of radiation oncologist and thoracic surgeon was organized to discuss the validity of the Markov model scenario, variables extracted through the systematic literature review, and the predicted Markov model results.

## RESULTS

### Predicted life expectancy and second-order Monte Carlo simulation

**Fig. 2.**
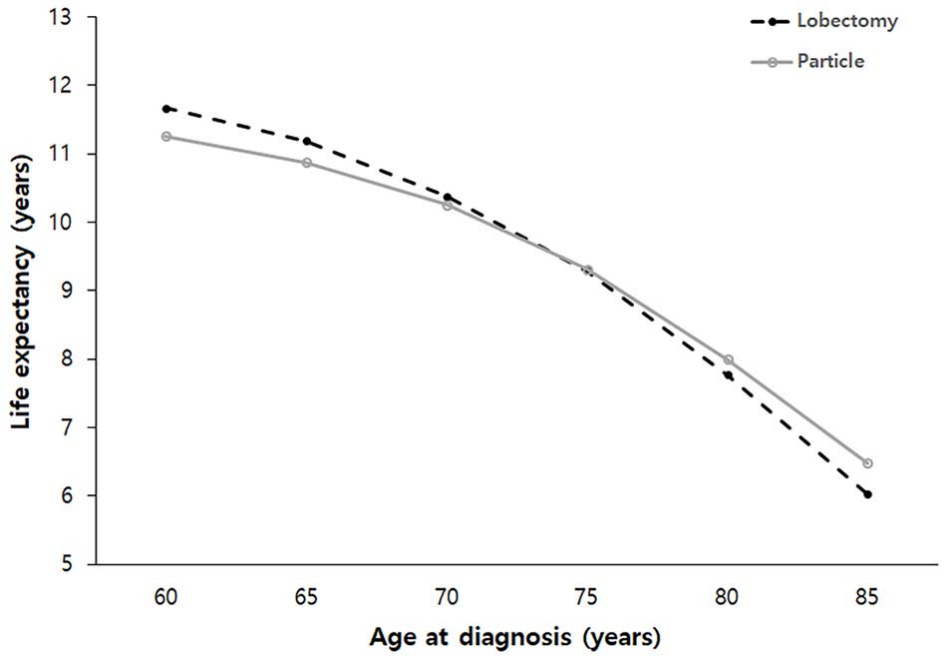
Estimated life expectancy stratified by age at diagnosis in patients with stage IA NSCLC after lobectomy or PBT. PBT; Particle beam therapy.

Life expectancies following lobectomy at 60, 65, 70, 75, 80 and 85 years of age at diagnosis were estimated to be 11.7, 11.2, 10.4, 9.3, 7.8 and 6.0 years, respectively. Life expectancies following PBT at 60, 65, 70, 75, 80 and 85 years of age at diagnosis were estimated to be 11.3, 10.9, 10.3, 9.3, 8.0 and 6.5 years, respectively. Estimated life expectancy curves for each cohort are illustrated in Fig. 2. The mean differences and 95% confidence intervals (CI) for estimated life expectancy difference between the lobectomy and PBT groups are listed in Table 2. The lobectomy group showed better life expectancy than that of the PBT group for patients under 70 years of age. The difference of life expectancy between the lobectomy and PBT groups was statistically insignificant at 70- ~ 75-years-old or over. The Markov model predicted the life expectancy of a 70-year-old patient who underwent lobectomy or PBT to be 10.4 and 10.3 years, respectively. The 95% CI for the difference in life expectancy between the lobectomy and PBT groups was −0.102 to 0.320 years at 70 years of age (*P* = 0.1377).

**Table 2:** Second-order Monte Carlo simulation stratified by age: difference in life expectancy

Age (years old)	Intervention	Life expectancy (years)				
		Estimation	Mean difference	95% CI		P value
60	Lobectomy	11.7	0.41308	0.2125	0.6115	0.000018
	PBT	11.3				
65	Lobectomy	11.2	0.30479	0.1030	0.5105	0.00164
	PBT	10.9				
70	Lobectomy	10.4	0.11637	−0.1020	0.3200	0.137768
	PBT	10.3				
75	Lobectomy	9.3	−0.03676	−0.2355	0.1555	0.359424
	PBT	9.3				
80	Lobectomy	7.8	−0.24334	−0.4310	−0.0740	0.003849
	PBT	8.0				
85	Lobectomy	6.0	−0.4514	−0.622	−0.2795	0.00001
	PBT	6.5				

### One-way and two-way sensitivity analyses

One-way sensitivity analysis was performed for the group of 70-year-old patients. The tornado diagram analysis indicated the most influential variables on life expectancy after lobectomy and PBT were the probability of disease progressions (Supplementary Fig. 1). Our model predicted that lobectomy was the preferred strategy for 70-year-old patients when variable values remained constant. However, the preferred strategy could shift to PBT if the probability of disease progression after lobectomy was >0.0462 (Supplementary Fig. 2A) or if the probability of disease progression after PBT was <0.0504 (Supplementary Fig. 2B).

Two-way sensitivity analysis was also performed for 70-year-old patients to determine the effect on life expectancy when the probability of disease progression and another intervention variable changed simultaneously after lobectomy or PBT (Supplementary Fig. 3). If the variables changed within the results of published studies as shown in Table 1, lobectomy and PBT were probabilistically similar with respect to being a preferred treatment option for patients at 70 years of age as the area of preferring lobectomy and PBT were similar.

**Fig. 3.**
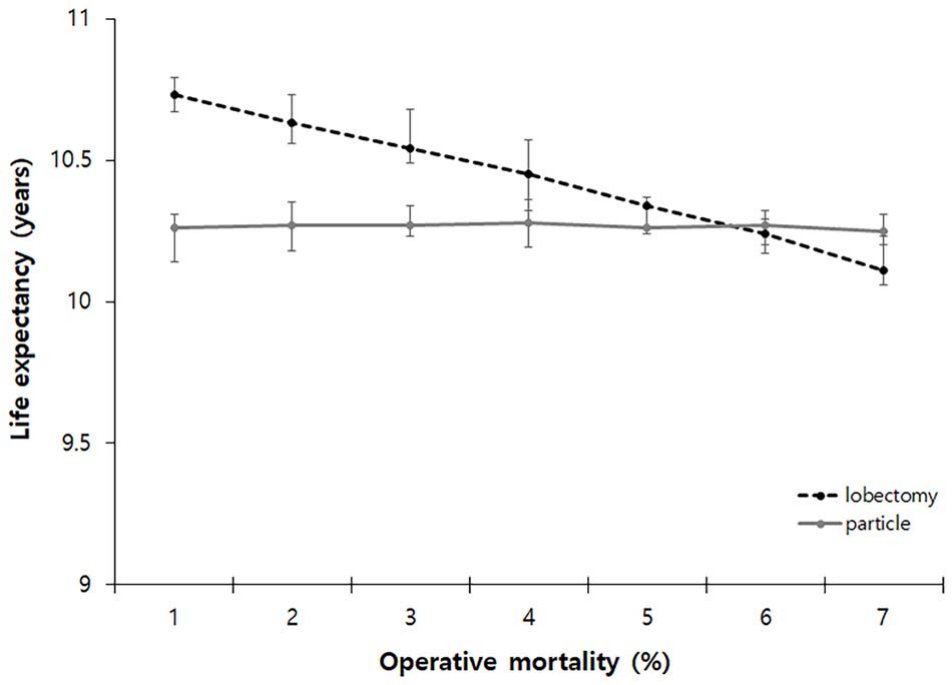
One-way sensitivity analysis of varying operative mortality after primary treatment at 70 years of age. The preferred strategy when the operative mortality was under 5%. However, the preferred strategy could be changed to PBT if the operative mortality after lobectomy was over 5%. PBT; Particle beam therapy.

A sensitivity analysis was also performed in 70-year-old patients to observe changes in life expectancy according to the changes in operative mortality. Our model predicted lobectomy was the preferred strategy when operative mortality was under 5%. However, the preferred strategy was changed to PBT if the operative mortality after lobectomy was over 5% (Fig. 3).

#### Model validity

**Fig. 4.**
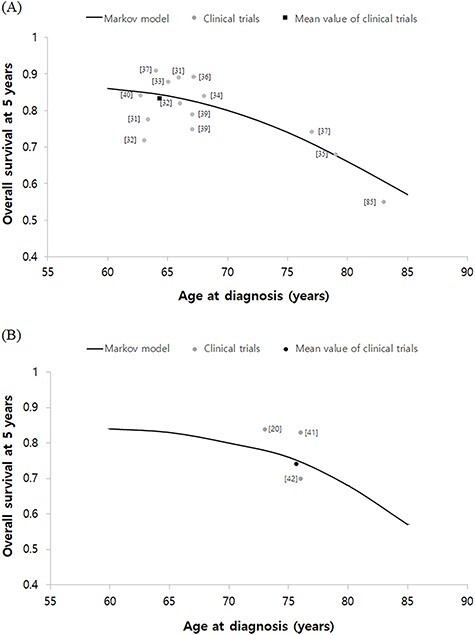
Validation of the Markov model. Predicted five years survival curve after lobectomy (A) and PBT (B) from our Markov model were shown. The gray circle dots represent the survival outcomes of real studies. In real studies, the median age of each cohort was set to a representative value of age at diagnosis. The black square dot is the average of these real studies. Overall survival of lobectomy and PBT were about 1% and 2% lower in the mean of real studies than those of the Markov model, respectively. PBT; Particle beam therapy.

The predicted five-year overall survival rate after lobectomy is illustrated in Fig. 4a. The five-year overall survival rates reported from the actual clinical studies are also presented and are indicated as gray dots [31–40]. For the evaluation using data from actual clinical studies, the representative value of age at the time of diagnosis was set as the median age of the patient group participating in the study. A black square in Fig. 4 indicates the average of these real studies of lobectomy and are positioned very close to the survival curve of our Markov model. The mean value of the five-year overall survival rate in the actual clinical studies showed only a 1% difference from the predicted value in our Markov model. The predicted five-year overall survival rate after PBT is illustrated in Fig. 4b. The five-year overall survival rates presented in the actual clinical studies are also shown and are indicated as gray dots [20, 41, 42]. Again, a black square dot shows the average of the real studies of PBT and are positioned very close to the survival curve of our Markov model. The mean value of the five-year overall survival rate in the actual clinical studies showed only a 2% difference from the predicted value in our Markov model.

## DISCUSSION

PBT is a radiotherapy technology capable of high-precision irradiation due to its special physical property called the Bragg peak and special carbon ion has greater relative biological effectiveness due to higher linear energy transfer compared to that of conventional radiotherapy using X-ray. Consequently, advances in PBT are expected to decrease toxicities and improve clinical outcomes for patients with early-stage NSCLC. Recently, excellent outcomes for PBT in stage IA NSCLC have been reported. The local control rate exceeds 95% in patients who underwent PBT in tumors less than 3 cm that are located in the peripheral lung, and serious side effects after treatment are rarely reported [19, 43–45]. As experience with PBT dramatically accumulates, Japanese groups have recently attempted to increase the treatment effect by reducing the fraction number of PBT using carbon ions. These investigators have tried to change the protocol from treatment with 60 Gy delivered in 4 fractions for two weeks to 50 Gy delivered as a single fraction in one day. In a dose escalation clinical trial of single-fraction carbon ion radiotherapy for peripheral stage I NSCLC, there were no primary tumor failures in small tumors 2 cm or less in size that receive 44 Gy relative biological effectiveness (RBE) or more [44].

Although it was not a PBT, three previous trials attempted to compare the results of SBRT with X-ray and lobectomy in patients with early stage NSCLC, the STARS trial (NCT00840749), the ROSEL trial (NCT00687986) and the ACOSOG Z4099 trial (NCT01336894); however, the trials were closed early due to slow accrual. Since then, SBRT has not yet been directly compared with lobectomy in any randomized trials. There have been several systemic reviews and meta-analysis studies, which all conclude that the survival rate is higher in the lobectomy group than that in the SBRT group [51–53]. However, there are some interesting results that have reported no statistical difference in survival rates between lobectomy and SBRT groups when the patient group is limited to those aged 75 years or older [51, 54]. The reason for these results is considered to be that operative mortality tends to increase with age. Recently, Stoke *et al.* reported that operative mortality increases significantly with age. Differences in post-treatment mortality between lobectomy and SBRT groups increase as a function of age, with the largest differences in favor of SBRT observed among patients older than 70 years of age [15]. These results from previous studies are consistent with the current results predicted using our Markov model. In our study, lobectomy showed excellent results in patients under 70 years of age, but there was no difference between the two groups for patients over 70 years of age (Table 2).

Operative mortality should be one of the most important considerations in determining the treatment modality for Stage IA NSCLC. In this study, for the sake of simplification of the model, a simple scenario was applied in which operative mortality simply increased as age of the patient increased. Operative mortality may generally increase with age, but in practice more diverse factors should be considered. Husain *et al.* reported findings that the Charlson-Deyo comorbidity score, which scores a patients’ underlying diseases with age, influences operative mortality [55]. Based on this, PBT may be advantageous if operative mortality increases due to an underlying disease, even for young patients, whereas lobectomy may be advantageous for patients with underlying diseases, even for older patients. In addition, several studies have reported operative mortality can be reduced and that there is no difference in disease control rates when minimally invasive surgery like VATS and RATS are performed rather than an open thoracotomy [38, 56, 57]. When determining a treatment modality, it should be considered which surgical technique will be used.

As noted previously, the advantage of lobectomy would be the survival gain obtained through adjuvant therapy due to finding subclinical metastases through mediastinal lymph node dissection. However, as operative mortality increases, there comes a point in which the survival gain through mediastinal lymph node dissection will be lost compared to that of PBT. In order to predict this transition point, we performed a sensitivity analysis that detected changes in life expectancy post lobectomy and post PBT when operative mortality was changed. As a result, when operative mortality exceeded 5% in 70-year-old patients, it was predicted there would be no statistical difference in life expectancy between patients undergoing lobectomy and those undergoing PBT. As there are still no randomized clinical trials, the result of our present study indicating the preferred strategy changes as the operative mortality changes with the crucial point being around 5% may be a useful reference indicator for a situation in which decisions need to be made in the clinical field.

There are several limitations in the current study. First, it is not an actual clinical trial, but instead a hypothetical comparative study using a Markov model. Therefore, accepting the results should be done with caution. However, we constructed scenarios as well as possible so the Markov model would reflect the actual clinical situations. In addition, we tried to extract variables for use in computer simulations from clinical studies with as high a level of evidence as possible. As a result, the survival rates predicted through the Markov model were not significantly different from the survival rates of actual clinical studies, which helped to validate our model (Fig. 4). Second, it was pointed out by members of the tumor board that according to the age reported by the Stoke *et al.* [15], the operative mortality used in the Markov model was set too high and was far from reality. For example, a randomized trial using a Japanese cohort (trial No. JCOG0802/WJOG4607L) compared segmentectomy and lobectomy for peripherally located NSCLCs of a diameter less than 2 cm with neither 30-day nor 90-day mortality being observed [58]. However, it is also true that there are many studies that report high mortality after lobectomy [15, 86, 87]. Operative mortality can be influenced by various factors such as race, underlying disease and surgeon skill. There is no known model that can accurately predict operative mortality in the clinical field. There would be various methods for clinicians to predict operative mortality. In this situation, if the operative mortality is calculated with only a few limited prognostic factors, the utilization of the Markov model would be degraded. The current study corrected this problem by developing a model that directly changed operative mortality (Fig. 3). Third, as the clinical results of PBT remain so scarce, it is possible the PBT variables used in our Markov model were based on limited clinical data and may not be fully representative. However, if there were many clinical results, it would be better to perform meta-analysis without having to do the Markov model analysis. The Markov model analysis was meaningful in that it tried to virtually predict the results of a randomized controlled trial in a situation where clinical data is insufficient and a condition in which it would be difficult to perform an actual randomized controlled trial.

In conclusion, for medically operable patients with peripherally located stage IA NSCLC, our Markov model verified the preferred strategy for lobectomy and PBT may vary with changes in operative mortality as it relates to age or comorbidity. As it is a comparative study using a virtual model, application of the results directly to clinical practice should be done cautiously. However, until future results from actual randomized controlled trials become available, we hope our current results will serve as a rational basis for clinicians to decide treatment modality for patients with stage IA NSCLC. In addition, we recommend that operative mortality and factors influencing it should be considered when designing future randomized studies.

## Supplementary Material

Supplementary_file_1_JRRS-D-21-00062_rrab060

Supplementary_file_2_A_and_B_JRRS-D-21-00062_rrab060

Supplementary_file_3_JRRS-D-21-00062_rrab060
