# Supplementary figures and images for: Virtual randomized study comparing lobectomy and particle beam therapy for clinical stage IA non-small cell lung cancer in operable patients
Source: J Radiat Res. 2021 Jul 5;62(5):884–93. doi: 10.1093/jrr/rrab060 (PMC8438263; doi:10.1093/jrr/rrab060)

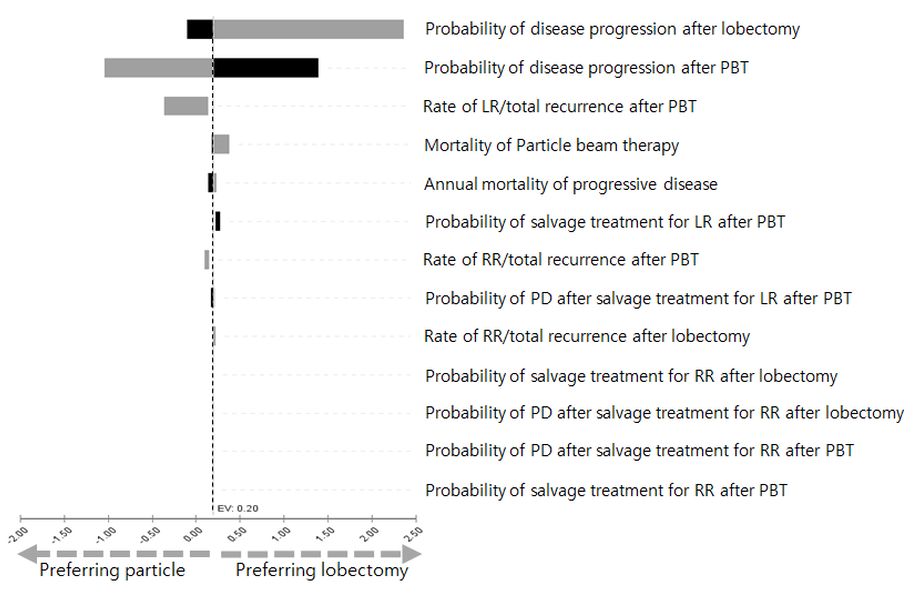

Supplement: Supplementary_file_1_JRRS-D-21-00062_rrab060 [file supplementary_file_1_jrrs-d-21-00062_rrab060.docx]

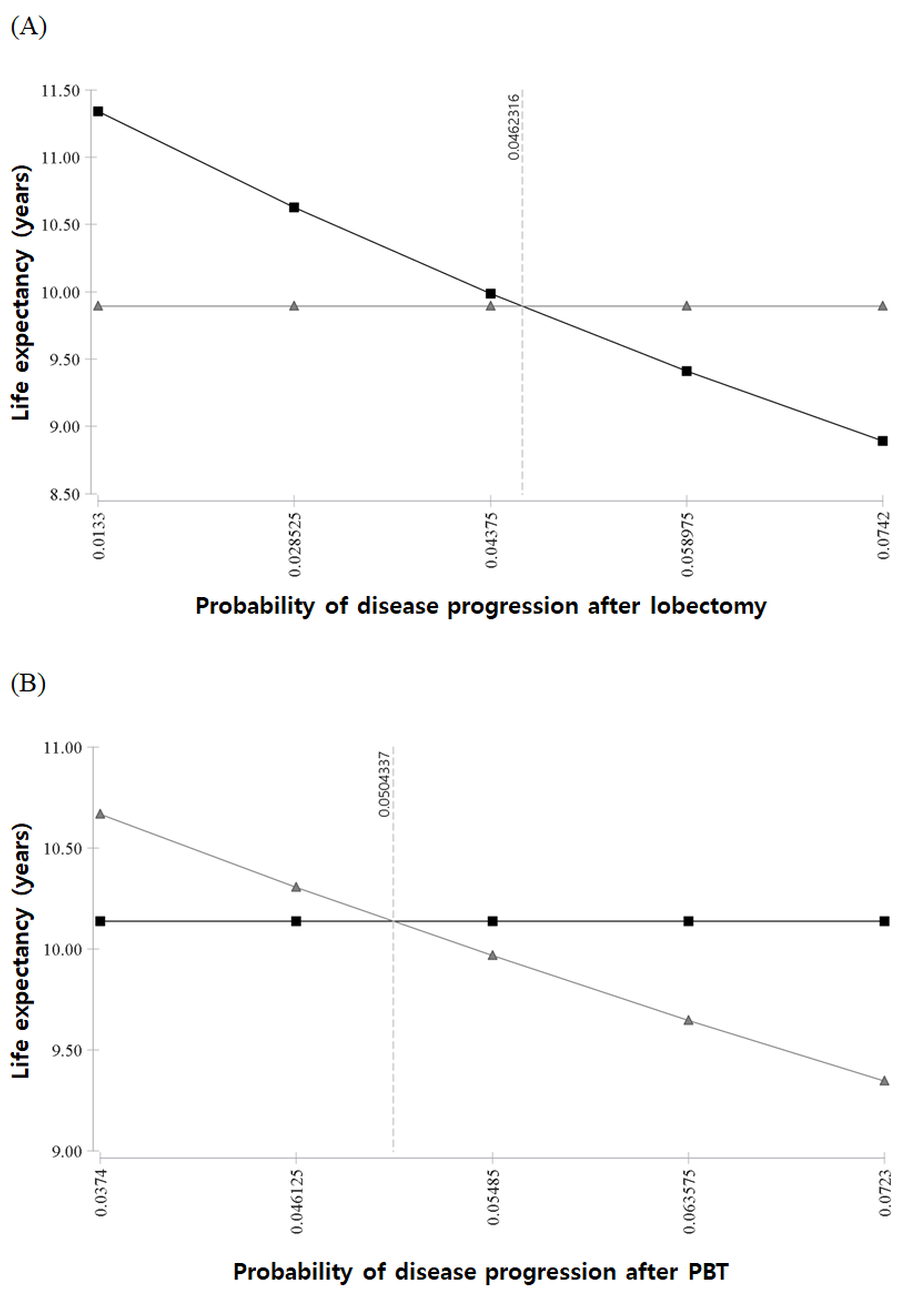

Supplement: Supplementary_file_2_A_and_B_JRRS-D-21-00062_rrab060 [file supplementary_file_2_a_and_b_jrrs-d-21-00062_rrab060.docx]

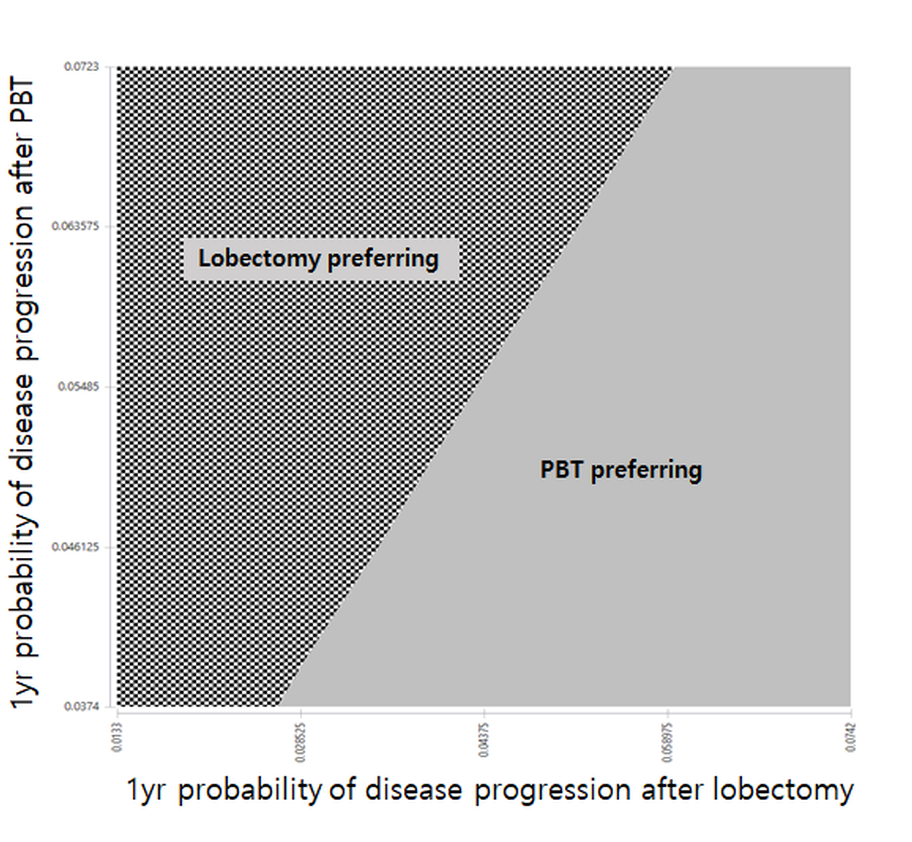

Supplement: Supplementary_file_3_JRRS-D-21-00062_rrab060 [file supplementary_file_3_jrrs-d-21-00062_rrab060.docx]
